# A Resilient Cloud–Edge Digital Twin Framework for Urban UAV Logistics Under 3D Blockages and ADS-B Signal Anomalies

**DOI:** 10.3390/s26123778

**Published:** 2026-06-13

**Authors:** Hanyang Tong, Yansheng Chen, Yilong Liu, Feige Huang, Jinlong Sun

**Affiliations:** School of Communications and Information Engineering, Nanjing University of Posts and Telecommunications, Nanjing 210003, China; b23012429@njupt.edu.cn (H.T.); 1224013833@njupt.edu.cn (Y.C.); 1224014141@njupt.edu.cn (Y.L.); 1225013633@njupt.edu.cn (F.H.)

**Keywords:** UAV logistics, cloud–edge digital twin, stochastic diffusion sampling, ADS-B signal anomalies, progressive distillation, trajectory optimization, time difference of arrival

## Abstract

Urban low-altitude unmanned aerial vehicle (UAV) logistics networks face critical operational bottlenecks due to complex three-dimensional spatial blockages, continuous communication diffraction, and severe vulnerability to information-layer threats such as Automatic Dependent Surveillance—Broadcast (ADS-B) signal anomalies. To address these interconnected challenges, this paper proposes an event-driven, cloud–edge collaborative digital twin framework to guarantee continuous multi-link communication and flight safety. The architecture operates through a dual-tier “Teacher–Student” paradigm. Under secure conditions, a cloud digital twin acts as a high-capacity “Teacher,” employing Density-Based Spatial Clustering of Applications with Noise (DBSCAN) to partition heterogeneous user topologies. It then utilizes an energy-guided stochastic diffusion sampling (EGSDS) method to refine initial macroscopic routing, generating precise, outage-free global trajectories by systematically minimizing non-line-of-sight (NLoS) observation penalties and kinematic regularization costs. To counteract signal anomalies, a distributed Time Difference of Arrival (TDOA) anchor network continuously validates UAV coordinate integrity. If a threshold is breached, control authority is instantly transferred to the UAV’s edge digital twin. This resource-constrained edge tier relies on a localized “Student” network trained via progressive distillation. By compressing the computationally heavy iterative diffusion process into a rapid one-step inference model, the UAV autonomously generates a secure, short-range emergency path that strictly adheres to minimum communication thresholds. Once interference clears, the cloud seamlessly regains control to complete the logistics mission. Experimental results demonstrate that the proposed scheme significantly outperforms conventional heuristic routing methods in cloud-based scenarios. Furthermore, the edge-based distillation mechanism substantially improves the overall trajectory survival rate under signal anomalies, ensuring resilient and continuous logistics operations.

## 1. Introduction

In recent years, thanks to the high maneuverability, flexible deployment capabilities, and precision, UAVs have been a key focus of research in urban low-altitude logistics networks [1]. These logistics networks integrate aviation platforms, multi-link communication, and autonomous control technologies, with the aim of enabling efficient “last-mile” delivery in smart cities [2]. Navigation and communication form the foundation of these networks. They leverage real-time, high-precision positioning to coordinate flight paths within three-dimensional airspace [3,4]. With the deployment of UAVs in dense urban environments, these systems now operate within highly heterogeneous 3D topologies. They face continuous physical blockages from high-rise buildings and complex urban canyons. The severe spatial diffraction and dynamic communication conditions challenge traditional routing paradigms.

To address this, many papers have explored evolutionary algorithm, swarm intelligence algorithm, and other optimization methods [5]. However, these algorithms are often highly complex and it is difficult to fully guarantee the operational safety. Cloud–Edge collaborative computing has gained attention as an effective approach. It forms the intelligent backbone of modern UAV networks. It enables low-latency, high-efficiency trajectory optimization and emergency decision-making by leveraging both edge and cloud computational capabilities [6,7]. Recent work shows that continuous data exchange facilitates reliable multi-link communication among ground users, transit stations, and cloud platforms [8]. Digital twin technology has also been incorporated into these logistics networks by many researchers to enable better real-time monitoring and visualization [9]. It enables a wide range of applications, including environmental reconstruction, dynamic trajectory planning, and threat mitigation [10]. A novel architecture named Heterogeneous UAV Digital Twin Network (HU-DTN) was proposed in [11]. A digital twin assisted path-planning framework for a UAV swarm with two phases: global path planning and real time trajectory planning was proposed in [12].

In addition, a novel energy consumption, throughput, and delay optimization framework that leverages Deep Reinforcement Learning with attention mechanisms to address the NP-hard combinatorial-continuous optimization challenge of jointly determining UAV visiting sequences was proposed in [13]. Recent architectures have also been designed to guarantee continuous multi-link communication and absolute flight safety against physical blockages and information-layer threats like Automatic Dependent Surveillance—Broadcast (ADS-B) spoofing [14,15].

In terms of research progress in algorithms, recent algorithms use adaptive AI like deep reinforcement learning (DRL) for dynamic environments. First, continuous trajectory optimization under jamming has been modeled using TD3 algorithms to improve throughput and flight stability [16]. Also, an enhanced deep reinforcement learning approach that encompasses two distinct learning stages, namely the reinforced and self-supervised, was proposed in [17]. Furthermore, within UAV-supported logistics communication systems, energy-efficient DRL-based methods [18] have become critical.

However, existing research still presents several critical limitations. First, most studies separate physical trajectory planning from information-layer security. They do not jointly address complex 3D urban blockages and malicious information-layer errors, such as ADS-B signal anomalies. Second, while traditional algorithms and standard deep learning models optimize parameters effectively, they require high computational power and extensive iterative processing. Therefore, they are difficult to deploy on resource-constrained UAVs. Third, many trajectory optimization frameworks rely on simplified binary line-of-sight communication models. These models fail to capture the continuous spatial diffraction penalties present in real-world urban canyons.

In complex urban environments, centralized trajectory planning is highly vulnerable to information-layer errors. These errors come from not only malicious ADS-B spoofing and jamming, but also physical factors like multipath fading and NLoS delays. Both types of anomalies cause the cloud digital twin to calculate routes based on incorrect UAV coordinates. To address these challenges, this paper proposes a novel event-driven, cloud–edge collaborative digital twin framework. This dual-tier architecture uses a TDOA-based verification mechanism to monitor coordinate safety. When a position error exceeds the safety threshold, the system triggers an automatic handoff to a lightweight edge model. This guarantees continuous communication and flight safety for urban UAV logistics, without needing to classify the exact source of the anomaly. The main contributions of this paper are summarized as follows:

1. To overcome the high computational complexity of joint trajectory planning and security optimization in dynamic environments, we propose a decoupled hierarchical optimization paradigm. By mapping the physical world into two asynchronously updated state spaces, this approach mathematically reformulates the joint macro-micro optimization bottleneck into an intensive cloud-based global routing task and a localized edge-based real-time survival response.
2. To accurately simulate the physical environment and address the inaccuracy of simplified binary blockage models in predicting signal attenuation, we introduce a continuous spatial diffraction loss model based on ITU-R Recommendation P.526. Current digital twin studies for UAV logistics often rely on simplified binary blockage models. In contrast, our approach more accurately captures signal propagation in urban canyons. This allows the system to precisely predict and synchronize multi-link communication quality within the digital space.
3. To solve the problem of maintaining communication connectivity while minimizing flight distance in heterogeneous urban topologies, we introduce a training-free Energy-Guided Stochastic Diffusion Sampling (EGSDS) mechanism for macro-scale trajectory refinement. Rather than training a neural network for score estimation, this method innovatively adapts Langevin dynamics to the physical context. It utilizes the digital twin’s precise 3D environmental gradients as an analytical energy field, systematically guiding the path to bypass severe NLoS diffraction zones without requiring large datasets.
4. To mitigate the threat of information-layer errors such as ADS-B signal anomalies under strict onboard constraints, we introduce an event-triggered authority handoff mechanism coupled with a progressive distillation network. This design breaks the blind reliance on information-layer telemetry via independent physical-layer radio frequency (RF) TDOA verification. Meanwhile, the progressive distillation structurally compresses the heavy iterative sampling process into a rapid one-step inference model, ensuring immediate evasive maneuvers under strict onboard hardware limits.

## 2. System Model and Problem Formulation

This section establishes a rigorous mathematical model for an urban low-altitude logistics network characterized by severe physical blockages and information uncertainty. The system accounts for the 3D topology of heterogeneous urban canyons, the diffraction attenuation of spatial multi-link communications, and the information asymmetry inherent in the digital twin architecture. Consequently, we formulate an event-driven cloud–edge collaborative framework constrained by flight kinematics, time windows, and dual safety thresholds.

### 2.1. Heterogeneous User-Driven 3D Urban Topology

Consider a rectangular urban region $R\in R^{2}$ with dimensions L×W. Within this region, there is a set of *K* user nodes, denoted by $P=\{U_{1},\ldots ,U_{K}\}$, requiring logistics delivery. The precise ground coordinates of each node are known and given by $U_{k}=\left(x_{k},y_{k},0\right)$. In reality, rather than being uniformly distributed, these users exhibit significant spatial clustering. They are distributed across sub-regions characterized by varying density attributes (e.g., extremely high-density clustering in central business districts (CBDs), moderate density in residential areas, and low-density dispersion in suburban or industrial zones).

To capture this spatial heterogeneity and reduce the complexity of large-scale scheduling, the system employs the DBSCAN (Density-Based Spatial Clustering of Applications with Noise) algorithm. Based on physical distances and local densities, it partitions the users P into *J* non-overlapping sub-regional clusters, denoted by $C=\{C_{1},\ldots ,C_{J}\}$. Within each identified sub-region $C_{j}$, a dedicated logistics transit station $T_{j}$ is deployed. The collection of all such transit stations forms the set $T=\{T_{1},\ldots ,T_{J}\}$. This station serves not only as a takeoff and landing pad for UAVs but also as a Time Difference of Arrival (TDOA) monitoring anchor node equipped with high-precision clock synchronization. Its coordinates are defined as $T_{j}=\left(x_{T_{j}},y_{T_{j}},H_{T_{j}}\right)$, where $H_{T_{j}}$ represents the height of the base station tower.

Once the clustering of users and relay stations is established, a set of $N_{b}$ high-rise buildings $B=\{B_{1},\ldots ,B_{N_{b}}\}$ is placed within the geographic boundary of each cluster $C_{j}$. This configuration faithfully reflects the physical reality of urban environments, where user nodes and buildings are densely interspersed [19,20]. Each building $B_{m}$ is modeled in three-dimensional space as a solid rectangular cuboid:

(1) $$B_{m}=\left\{x_{m},y_{m},w_{m},l_{m},h_{m}\right\},\;\forall m\in \left\{1,2,\ldots ,N_{b}\right\}$$

where $\left(x_{m},y_{m}\right)$ denotes the central ground coordinates of the *m*-th building, and $w_{m},l_{m},$ and $h_{m}$ represent its width, length, and height, respectively.

To enable global coordination, a cloud center with substantial computational capacity is deployed within the region R. To minimize the deployment footprint and leverage existing urban infrastructure, the Cloud DT is integrated into the structural apex of the tallest building within the region. Its coordinates are defined by the highest building $B_{max}\in B$, such that $M_{cloud}=\left(x_{m^{*}},y_{m^{*}},h_{m^{*}}\right)$, where $m^{*}=\arg\max_{m}\left\{h_{m}\right\}$. This strategic placement ensures a commanding overhead perspective.

One dedicated UAV is assigned to each cluster, with the full fleet defined as the set V. At time step *t*, the three-dimensional position of the UAV serving cluster *j* is denoted by $V_{j}\left(t\right)=\left(x_{v_{j}}\left(t\right),y_{v_{j}}\left(t\right),H_{V}\right)$. During flight, each UAV must simultaneously maintain three spatial communication links: one with the ground-level user $U_{k}$, one with the relay station $T_{j}$, and one with the global cloud center $M_{cloud}$.

### 2.2. Cloud–Edge Collaborative Digital Twin Architecture

To enable reliable scheduling in a highly dynamic environment subject to potential interference, the system adopts a Dual-tier Cloud–Edge Digital Twin architecture, similar to [21]. This architecture maps the physical world onto two asynchronously updated state spaces: a cloud tier responsible for macro-level coordination, and an edge tier dedicated to emergency collision avoidance.

Leveraging substantial computational resources, the cloud digital twin maintains the global topology and performs prior generation of complex trajectories. At time *t*, the cloud DT state tuple is defined as:

(2) $$DT_{Cloud}\left(t\right)=\left\{B,\;P,\;T,\;V^{\mathrm{obs}}\left(t\right),\;\Theta_{cloud}\right\}$$

where $\Theta_{cloud}$ denotes the high-precision global 3D outage-avoidance and delivery trajectory pre-computed and dispatched by the cloud, based on the global 3D obstacle topology and communication heat map; and $V^{obs}\left(t\right)$ denotes the set of UAV observation states received by the cloud.

Consequently, an unknown spatiotemporal error matrix $E_{j}\left(t\right)$ exists between the observation state $V_{j}^{obs}\left(t\right)$ available to the cloud and the true state $V_{j}^{true}\left(t\right)$ [22]:

(3) $$V_{j}^{\mathrm{obs}}\left(t\right)=V_{j}^{\mathrm{true}}\left(t\right)⊕E_{j}\left(t\right)$$

where ⊕ denotes the state corruption operator, representing the integration of sensory noise, communication delay, or malicious spatial spoofing onto the true physical coordinates [23]. When $E_{j}\left(t\right)\neq 0$, the cloud’s high-precision outage-avoidance commands, which are computed based on corrupted coordinates, become misleading rather than corrective.

To guard against the risk of catastrophic communication blackouts caused by information-layer attacks, a lightweight edge digital twin dedicated to Emergency Fallback is deployed onboard UAV *j*:

(4) $$DT_{Edge,j}\left(t\right)=\left\{V_{j}^{\mathrm{true}}\left(t\right),\;B_{local}\left(t\right),\;U_{local}^{\mathrm{emerge}}\left(t\right)\right\}$$

where $V_{j}^{true}\left(t\right)$ is the absolute and trustworthy pose obtained from the onboard high-precision BeiDou receiver; $B_{local}\left(t\right)$ is the local 3D building geometry perceived by onboard sensors; and $U_{local}^{emerge}\left(t\right)$ denotes the local emergency trajectory independently generated by the edge tier under crisis conditions.

A dynamic Boolean control flag $C_{flag}^{\left(j\right)}\left(t\right)\in \left\{0,1\right\}$ is introduced to define the boundary of authority between the two tiers:

1. $C_{flag}^{\left(j\right)}\left(t\right)=0$: Normal cloud control. The UAV follows the high-quality 3D outage-avoidance trajectory $\Theta_{cloud}^{*}\left(t\right)$ dispatched by the cloud.
2. $C_{flag}^{\left(j\right)}\left(t\right)=1$: Emergency edge takeover. The corrupted cloud signaling is severed, and $DT_{Edge,j}$ is activated to independently execute the survival trajectory $U_{local}^{emerge}\left(t\right)$.

### 2.3. Multi-Link Communication Model and 3D Spatial Blockage

To accurately capture the signal attenuation caused by urban canyon effects, this model incorporates continuous spatial diffraction loss based on ITU-R Recommendation P.526, replacing the conventional binary line-of-sight blocking model [24].

For the direct ray $L_{V,N}$($N\in \{U_{k},T_{j},M_{cloud}\}$), let $h_{m}$ denote the vertical distance by which the top edge of a building intrudes into the ray path. The Fresnel–Kirchhoff diffraction parameter $\nu_{m}$ is defined as [25]:

(5) $$\nu_{m}=h_{m}\cdot \sqrt{\frac{2(d_{1}+d_{2})}{\lambda \cdot d_{1}\cdot d_{2}}}$$

where $d_{1}$ and $d_{2}$ are the distances from the building edge to the transmitter and receiver, respectively, and λ is the signal wavelength.

The single-knife-edge diffraction loss (dB) induced by the building is expressed through a continuously differentiable approximation [26]:

(6) $$J\left(\nu_{m}\right)=\left\{\begin{array}{cc} 6.9+20\log_{10}\left(\sqrt{{\left(\nu_{m}-0.1\right)}^{2}+1}+\nu_{m}-0.1\right), & \nu_{m}>-0.78\\ 0, & \nu_{m}\leq -0.78 \end{array}\right.$$

When multiple buildings obstruct the propagation path, calculating the exact recursive interference is computationally prohibitive for real-time digital twin synchronization. Therefore, we select the dominant obstacle causing the maximum attenuation to represent the spatial diffraction penalty for the link [27], which is defined as $J_{V,N}\left(t\right)=\max_{m}\left\{J\left(\nu_{m}\right)\right\}$.

To facilitate subsequent power calculations in the linear domain, we convert this logarithmic attenuation penalty into an additional path loss coefficient $\eta_{loss}^{V,N}\left(t\right)$:

(7) $$\eta_{loss}^{V,N}\left(t\right)=10^{\frac{-J_{V,N}\left(t\right)}{10}}$$

Based on the continuous diffraction penalty model described above, we can accurately calculate the instantaneous channel attenuation of the three key links for a UAV at any spatial point. Let $\beta_{0}$ denote the free-space channel power gain at a reference distance of 1 m.

It should be noted that every spatial link in this system is inherently bidirectional, as the UAV both transmits data to and receives commands from each of the three nodes ($U_{k}$, $T_{j}$, and $M_{cloud}$). Under the principle of channel reciprocity, the uplink and downlink share identical propagation geometry and diffraction environments; thus, the path loss coefficient $\eta_{loss}^{V,N}\left(t\right)$ applies equally to both directions. Nevertheless, the transmit powers of the UAV ($P_{V}$), the ground user ($P_{k}$), the transit station ($P_{T}$), and the cloud macro base station ($P_{M}$) are intrinsically distinct, satisfying $P_{M}\gg P_{T}>P_{k}$. Consequently, the uplink and downlink SNRs of each link exhibit asymmetry and must be modeled independently.

UAV-to-User Link. Let $D_{U}\left(t\right)={∥V_{j}\left(t\right)-U_{k}∥}_{2}$ be the three-dimensional Euclidean distance between the UAV and the ground user. The downlink SNR, representing the UAV transmitting payload data to the user, is given by [28]:

(8) $${\mathrm{SNR}}_{U}^{dl}\left(t\right)=\frac{P_{V}\cdot \frac{\beta_{0}}{D_{U}^{2}\left(t\right)}\cdot \eta_{loss}^{V,U}\left(t\right)}{\sigma^{2}}$$

where $\sigma^{2}$ denotes the additive white Gaussian noise (AWGN) power at the receiver. Conversely, the uplink SNR, representing the user transmitting its positional reports and acknowledgment signals back to the UAV is:

(9) $${\mathrm{SNR}}_{U}^{ul}\left(t\right)=\frac{P_{k}\cdot \frac{\beta_{0}}{D_{U}^{2}\left(t\right)}\cdot \eta_{loss}^{V,U}\left(t\right)}{\sigma^{2}}$$

Under a half-duplex operational assumption, the effective bidirectional communication capability of this link is bounded by the weaker of the two directions:

(10) $${\mathrm{SNR}}_{link,U}\left(t\right)=\min\left({\mathrm{SNR}}_{U}^{dl}\left(t\right),\;{\mathrm{SNR}}_{U}^{ul}\left(t\right)\right)$$

UAV-to-Transfer Station Link and UAV-to-Cloud Macro BS Link. By analogy with the UAV-to-User link, the bidirectional SNRs for the remaining two links are formulated in the same manner. Let $D_{T}\left(t\right)={∥V_{j}\left(t\right)-T_{j}∥}_{2}$ and $D_{M}\left(t\right)={∥V_{j}\left(t\right)-M_{cloud}∥}_{2}$ denote the respective Euclidean distances. The corresponding effective link SNRs are:

(11) $${\mathrm{SNR}}_{link,T}\left(t\right)=\min\left(\frac{P_{V}\cdot \frac{\beta_{0}}{D_{T}^{2}\left(t\right)}\cdot \eta_{loss}^{V,T}\left(t\right)}{\sigma^{2}},\;\frac{P_{T}\cdot \frac{\beta_{0}}{D_{T}^{2}\left(t\right)}\cdot \eta_{loss}^{V,T}\left(t\right)}{\sigma^{2}}\right)$$

(12) $${\mathrm{SNR}}_{link,M}\left(t\right)=\min\left(\frac{P_{M}\cdot \frac{\beta_{0}}{D_{M}^{2}\left(t\right)}\cdot \eta_{loss}^{V,M}\left(t\right)}{\sigma^{2}},\;\frac{P_{V}\cdot \frac{\beta_{0}}{D_{M}^{2}\left(t\right)}\cdot \eta_{loss}^{V,M}\left(t\right)}{\sigma^{2}}\right)$$

In all the expressions above, $\sigma^{2}$ denotes the additive white Gaussian noise (AWGN) power.

To ensure real-time data synchronization and command dispatch within the digital twin architecture, the UAV must sustain connectivity across all three links simultaneously. The spatial joint communication penalty field $H_{joint}\left(V_{j}\left(t\right)\right)$ is defined as the worst-case bidirectional link quality among the three, capturing the system-level bottleneck effect:

(13) $$H_{joint}\left(V_{j}\left(t\right)\right)=\min\left(SNR_{U}\left(t\right),\;SNR_{T}\left(t\right),\;SNR_{M}\left(t\right)\right)$$

To ensure the digital twin receives real-time updates, the system must meet a minimum data throughput requirement. We use Shannon’s theorem to link the large-scale joint SNR to the system capacity. The bidirectional joint throughput is:

(14) $$TH_{joint}\left(V_{j}\left(t\right)\right)=B\log_{2}\left(1+H_{joint}\left(V_{j}\left(t\right)\right)\right)$$

where *B* is the channel bandwidth. For safe control and steady data flow, this throughput must exceed the threshold: $TH_{joint}\left(V_{j}\left(t\right)\right)\geq TH_{th}$.

### 2.4. Flight Kinematic and Security Constraints

Within any continuous time interval Δt, the UAV $V_{j}\left(t\right)$ assigned to the *j*-th sub-region must adhere to the maximum cruising speed $v_{max}$ constrained by its rotor aerodynamics. The kinematic boundary of its discrete trajectory is strictly defined as:

(15) $${∥V_{j}\left(t+\Delta t\right)-V_{j}\left(t\right)∥}_{2}\leq v_{\max}\cdot \Delta t$$

To guarantee physical safety within the complex 3D urban topology, the system enforces a vertical clearance constraint. The UAV’s operational altitude $H_{V}$ must be greater than the highest building within the entire geographical region. This altitude configuration aligns with industrial low-altitude spatial planning designed to isolate unmanned traffic from dense ground structures. Additionally, cruising above the building canopy avoids the frequent vertical maneuvering required within tightly packed building canyons, thereby improving energy efficiency during forward flight.

Since multiple UAVs operate concurrently across different clusters, a horizontal separation constraint is required to prevent inter-UAV collisions, especially near cluster boundaries. For any two distinct UAVs m,n cruising at the same altitude layer, their spatial distance must exceed a safe separation threshold $D_{safe}$ to avoid physical interference:

(16) $${∥V_{m}\left(t\right)-V_{n}\left(t\right)∥}_{2}\geq D_{safe},\;\forall t\in \left[0,T_{max}\right]$$

Furthermore, operating as a logistics system, the UAV must depart from the local transit station $T_{j}$, visit all assigned user nodes $P_{j}$ within the cluster, and safely return to $T_{j}$. This entire mission must be executed within the limited battery endurance and the maximum task time threshold $T_{max}$ [29,30].

Beyond physical limitations, the system must account for severe information-layer vulnerabilities. Specifically, this model addresses the vulnerability of the ADS-B protocol to spoofing and delay-forwarding attacks in low-altitude networks [31]. To monitor this, the system integrates the set of regional logistics transit stations *T* into a distributed monitoring network.

The control authority of the UAV is governed by a dynamic Boolean control flag $C_{flag}^{\left(j\right)}\left(t\right)\in \left\{0,1\right\}$. Under secure conditions, the UAV executes the global macroscopic cruising commands issued by the Cloud Digital Twin. However, if the signal anomalies deviation exceeds a critical safety threshold $D_{th}$, the system triggers the highest-level alert, terminating cloud commands and activating the Edge Digital Twin to take over the UAV. The mathematical formulation of this detection and handoff mechanism is detailed below.

In our framework, the handling of ADS-B signal anomalies is event-driven. In urban environments, an anomaly may originate from different sources, such as malicious spoofing or severe natural multipath fading. Our system does not focus on classifying the specific origins of these anomalies or building a specific stochastic attack model. Instead, it focuses on the resulting event: a spatial deviation between the reported coordinates and the TDOA-verified coordinates. The system manages these events using the dynamic safety threshold $D_{th}$. The system’s performance is evaluated through the false alarm rate and detection rate, which serve as the false positive and false negative analysis, as discussed in Section 4.

### 2.5. Problem Formulation

In the cloud-based route planning, based on the requirements of real-world logistics scenarios, we aim to minimize the overall task duration within each cluster while ensuring uninterrupted communication. The optimization problem (P1) can be formulated as:

(17) $$\left(P1\right):\min_{T_{j},\Theta_{cloud}}T_{total\_j}\;$$

(18) $$\begin{array}{cc} \; & s.t.\;TH_{joint}\left(V_{j}\left(t\right)\right)\geq TH_{th}\\ \; & \;\;\;T_{total\_j}\leq T_{\max} \end{array}$$

where $T_{total\_j}$ means total flight time of the UAV in *j*-th cluster. In practical logistics scenarios, UAV operations are strictly bounded by onboard battery capacity. To support the claim that minimizing the kinematic regularization cost $C_{kin}$ inherently translates to energy efficiency, we formulate a quantitative energy consumption model for rotary-wing UAV operations. The total battery depletion $E_{total}$ comprises propulsion energy and communication energy. The propulsion power required for a UAV flying at a forward speed *v* is modeled based on standard aerodynamic principles:

(19) $$P_{prop}\left(v\right)=P_{0}\left(1+\frac{3v^{2}}{U_{tip}^{2}}\right)+P_{i}{\left(\sqrt{1+\frac{v^{4}}{4v_{0}^{4}}}-\frac{v^{2}}{2v_{0}^{2}}\right)}^{1/2}+\frac{1}{2}d_{0}\rho sAv^{3}$$

where $P_{0}$ and $P_{i}$ are the blade profile power and induced power in the hovering state, respectively. $U_{tip}$ represents the tip speed of the rotor blade, and $v_{0}$ is the mean rotor induced velocity in hover. When the UAV hovers at local transit stations $T_{j}$ or executes sudden authority handoffs during TDOA anomaly verifications, the velocity v=0, yielding the hovering power $P_{hover}=P_{0}+P_{i}$.

Furthermore, the communication power $P_{comm}$ accounts for spatial data transmission across the multi-link network and onboard circuit consumption. Because the UAV in our framework is configured to maintain a constant cruising altitude $H_{V}$ above building canopies and operates at a steady maximum cruising speed $v_{max}$, the complex non-linear propulsion power simplifies to a constant $P_{prop}\left(v_{max}\right)$. Consequently, the relationship between the kinematic route length $D_{flight}$, the total mission duration $T_{total\_j}$, and actual battery depletion can be linearly bounded:

(20) $$E_{total}=P_{prop}\left(v_{max}\right)\frac{D_{flight}}{v_{max}}+P_{hover}T_{hover}+P_{comm}T_{total\_j}\leq E_{cap}$$

where $E_{cap}$ is the absolute battery capacity limit. This explicit analytical model mathematically bridges our objective function—minimizing the kinematic trajectory length and task duration—directly to the physical battery depletion. It validates that the outage-free paths generated by the EGSDS method safely adhere to the endurance limits without requiring a separate energy optimization layer.

**Figure 1:**
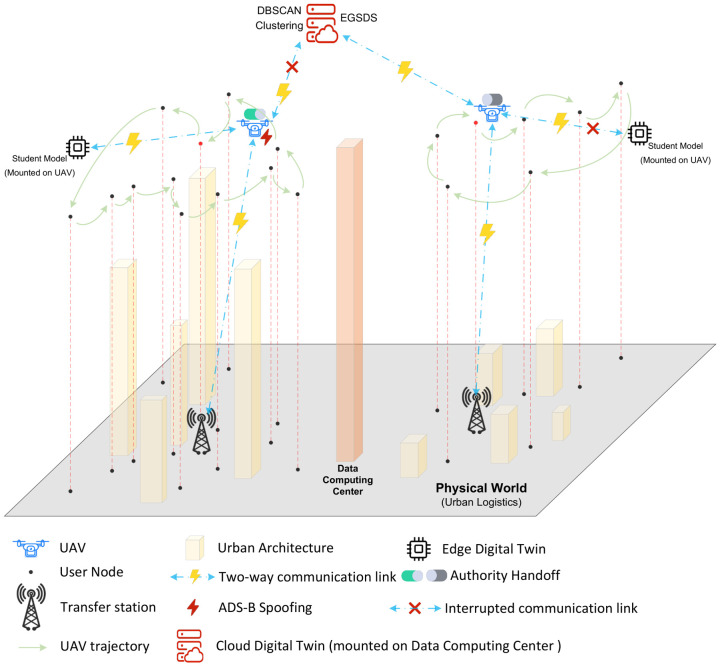
The overall architecture of the proposed cloud–edge collaborative digital twin framework for urban UAV logistics.

Figure 1 illustrates the architecture of the proposed cloud–edge collaborative framework. The architecture comprises a cloud tier for macroscopic coordination and an edge tier for autonomous evasion: the Cloud DT performs DBSCAN clustering and diffusion-based trajectory optimization, while the Edge DT facilitates rapid survival responses via distilled student models. Upon detecting ADS-B signal anomalies through the TDOA network, the system triggers an authority handoff, allowing the edge to instantly execute emergency evasion and ensure operational resilience under information-layer threats.

## 3. Cloud–Edge Collaborative Trajectory Optimization: From Teacher-Driven Routing to Student-Distilled Takeover

Solving the formulated mixed-integer non-linear programming (MINLP) problem (P1) is computationally intractable due to the NP-hard nature of combinatorial sequence routing coupled with continuous 3D communication outage avoidance. To ensure operational feasibility under limited UAV onboard resources, a structural engineering decoupling is fundamentally necessary.

The Cloud DT possesses substantial computational capacity to resolve the non-convex geometry of 3D urban environments using the iterative, gradient-based EGSDS algorithm. However, this centralized cloud optimization inherently relies on the information layer for telemetry, leaving it vulnerable to ADS-B signal anomalies or deep fading that corrupts coordinate synchronization. To prevent the cloud from issuing misleading trajectories during such information-layer crises, an independent physical-layer verification is required.

Hence, we integrate a distributed TDOA network to monitor physical signal arrival differences. Once a trust breach is detected, the system executes a hard authority handoff to the Edge DT. Because the resource-constrained UAV cannot execute the multi-step iterative optimization of the cloud during a crisis, we employ offline progressive distillation to compress the complex evasion logic into a lightweight student model. This integrated architecture establishes a resilient closed loop: the cloud maximizes mission economy under secure conditions, and the edge guarantees physical survival during information-layer failures.

In this architecture, “Cloud/Edge” and “Teacher/Student” represent a direct mapping viewed from two perspectives. “Cloud/Edge” defines the physical hardware deployment. Correspondingly, “Teacher/Student” defines the algorithmic knowledge transfer: the Cloud DT “Teacher” executes the complex multi-step EGSDS algorithm, while the Edge DT “Student” uses progressive distillation to compress this logic into a rapid, one-step inference model for onboard execution.

### 3.1. Macro-Scale Trajectory Optimization: Sequence Disentanglement

In realistic low-altitude urban logistics, user demands P are rarely distributed uniformly across the global region R. Instead, they exhibit significant spatial heterogeneity, often clustering densely in commercial business districts (CBDs) or residential blocks. Assigning a single trajectory to traverse all scattered users globally is strictly prohibited by UAV battery constraints and causes an exponential explosion in sequence optimization complexity.

**Figure 2:**
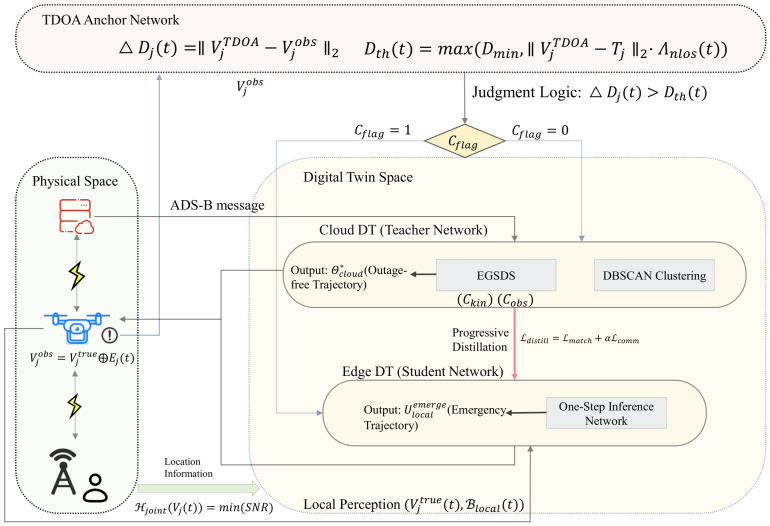
The dual-tier cloud–edge digital twin architecture.

Therefore, it is necessary to partition the global routing problem into localized, manageable sub-tasks. By evaluating the spatial proximity of user coordinates $U_{k}$, the Cloud DT employs the Density-Based Spatial Clustering of Applications with Noise (DBSCAN) algorithm, as depicted in the Cloud DT tier of Figure 2. This approach automatically groups geographically dense users into *J* independent clusters $C=\{C_{1},\ldots ,C_{J}\}$. For each identified cluster $C_{j}$, a local transit station $T_{j}$ is optimally deployed to serve as both the logistical takeoff hub and the TDOA monitoring anchor. This density-based topological division strictly bounds the flight range of each assigned UAV within its endurance limits $T_{max}$.

Once the localized user sets $P_{j}$ and their respective hubs $T_{j}$ are defined, the Cloud DT must establish a preliminary visiting order. Finding the initial optimal path is simply a shortest-path traveling salesman problem (TSP), which can be easily solved.

### 3.2. Trajectory Refinement via Energy-Guided Stochastic Diffusion Sampling

Although the TSP sequencing before solves the macroscopic routing problem, it generates straight-line flight segments between user nodes. These straight lines ignore the 3D buildings and communication blockages. If the UAV flies directly along these lines, it will likely enter severe Non-Line-of-Sight (NLoS) regions, leading to communication outages.

To solve this, the Cloud DT uses an Energy-Guided Stochastic Diffusion Sampling (EGSDS) method to reshape these risky straight lines. Unlike standard generative diffusion models that require large datasets to train a neural network for score estimation, our digital twin system possesses the exact 3D topology. Therefore, we model the trajectory refinement as a training-free Langevin dynamics process guided by an analytical physical energy field. To further clarify this design choice, it is helpful to distinguish the proposed EGSDS from recent data-driven diffusion trajectory planners. Conventional diffusion planners typically rely on deep neural networks trained offline on large expert demonstration datasets. Consequently, they often face challenges regarding performance degradation when deployed in unfamiliar or dynamic urban environments. In comparison, EGSDS is designed as a training-free optimization process. Because the Cloud DT provides precise 3D physical gradients as an analytical energy field, EGSDS reduces the reliance on historical data. This physics-guided approach helps the generated waypoints meet communication constraints without the need for extensive offline training, thereby improving environmental adaptability. Therefore, a direct numerical comparison with data-driven generative models is not the primary focus of this study, as EGSDS mainly aims at real-time physical convergence rather than statistical pattern matching.

By discretizing the continuous flight path into $K_{global}$ sequential waypoints, the trajectory is defined as:

(21) $$\Theta =\left\{V_{j}^{\left(1\right)},\;V_{j}^{\left(2\right)},\;\ldots ,\;V_{j}^{\left(K_{global}\right)}\right\}$$

The start node and end node $V_{j}^{\left(1\right)}=V_{j}^{\left(K_{global}\right)}=T_{j}$ act as fixed boundary conditions.

The total cost of a trajectory, denoted as $C_{total}\left(\Theta \right)$, measures its potential risk and deviation. We divide this cost into two mathematical components:

1. Observation Penalty Cost ($C_{obs}$): This term prevents the UAV from entering communication dead zones. Based on the joint communication quality $H_{joint}$, we define a tolerance-aware penalty:
  (22) $$C_{obs}=\lambda_{obs}\sum_{k=1}^{K_{global}}\max{\left\{0,\;\gamma_{alert}-H_{joint}\left(V_{j}^{\left(K_{global}\right)}\right)\right\}}^{2}$$
  Here, $\gamma_{alert}\in \left(0,1\right)$ is a predefined safety alert threshold, and $\lambda_{obs}$ is a large penalty weight. This function is non-linear: it only generates a penalty when the communication quality drops below $\gamma_{alert}$. If the UAV remains in a safe area, this cost is zero.
2. Kinematic Regularization Cost ($C_{kin}$): To ensure the trajectory is smooth and dynamically feasible for the UAV, we minimize the distance between consecutive waypoints:
  (23) $$C_{kin}=\lambda_{kin}\sum_{k=1}^{K_{global}-1}{∥V_{j}^{\left(k+1\right)}-V_{j}^{\left(k\right)}∥}^{2}$$
  Here, $\lambda_{kin}$ is a weight parameter controlling the smoothness. This term acts like a virtual elastic band, preventing unnecessary detours and reducing overall flight time.

The total cost $E\left(\Theta \right)=C_{obs}\left(\Theta \right)+C_{kin}\left(\Theta \right)$ acts as the exact physical energy field. In this cost formulation, the parameters $\lambda_{obs}$, $\lambda_{kin}$, and $\gamma_{alert}$ influence the trajectory optimization. Specifically, the weights $\lambda_{obs}$ and $\lambda_{kin}$ balance communication safety and flight distance. A larger $\lambda_{obs}$ guides the trajectory further away from spatial diffraction zones, while a larger $\lambda_{kin}$ results in a shorter flight path. Furthermore, the alert threshold $\gamma_{alert}$ affects the sensitivity to communication degradation. A larger $\gamma_{alert}$ causes the system to penalize the path earlier when the UAV approaches high-rise buildings. We treat the initial TSP straight line as the starting noisy state $\Theta_{0}$. Over $N_{iter}$ iterations, the path is refined through a stochastic gradient descent process. At each step *i*, the trajectory is updated using the exact physical gradient combined with an annealed Gaussian noise injection to escape local optima:

(24) $$\Theta_{i-1}=\Theta_{i}-\eta \nabla_{\Theta }E\left(\Theta_{i}\right)+\sigma_{i}z$$

where η is the learning rate, z∼N(0,I) is the standard Gaussian noise, and $\sigma_{i}$ is a linearly decaying noise schedule controlled by the iteration step. Physically, the term $\nabla_{\Theta }E\left(\Theta_{i}\right)$ systematically pushes the waypoints away from the severe diffraction zones directly above 3D buildings, while the injected noise $\sigma_{i}z$ ensures exploration. After $N_{iter}$ iterations, the model outputs the final, outage-free global trajectory $\Theta_{cloud}^{*}$.

It is worth noting that in practical deployments, certain user nodes may be inherently located within zones of severe signal attenuation. If the Cloud Digital Twin anticipates that the UAV’s subsequent target resides in a high-risk communication region, it proactively modifies the flight profile. To prevent telemetry outages, the Cloud DT commands the UAV to increase its cruising altitude as it approaches the hazardous zone, thereby mitigating spatial diffraction. Once positioned directly above the target node, the UAV executes a strict vertical descent to safely complete the delivery.

In 3D urban environments, the trajectory optimization space is inherently non-convex, precluding a closed-form global optimality guarantee. Nevertheless, the convergence behavior and stability of EGSDS are structurally ensured by the design of its annealed Langevin dynamics, which we discuss along five dimensions.

Existence of stationary solutions: The total energy $E\left(\Theta \right)=C_{\mathrm{obs}}\left(\Theta \right)+C_{\mathrm{kin}}\left(\Theta \right)$ is non-negative by construction, and the feasible trajectory space is compact due to the bounded physical domain [0,L]×[0,W]. By the extreme value theorem, E(Θ) necessarily attains its infimum within this space, guaranteeing the existence of at least one stationary solution $\Theta^{*}$.

Convergence of the iterative process: The deterministic gradient term $-\eta \nabla_{\Theta }E\left(\Theta_{i}\right)$ drives waypoints monotonically toward lower-energy configurations at each step, consistent with the standard descent property of gradient-based methods. This descent behavior, inherent to the Langevin dynamics structure, provides the iterative convergence of the optimization process.

Robustness to initialization: The stochastic noise $\sigma_{i}z$ injected in early iterations endows the process with broad exploration capability, enabling trajectories to escape shallow local minima induced by building blockages. This substantially reduces sensitivity to the TSP straight-line initialization $\Theta_{0}$, as diverse initial configurations are effectively reachable during early-stage sampling.

Stability under stochastic perturbations: As the noise schedule $\sigma_{i}=\sigma_{0}\left(1-i/N_{\mathrm{iter}}\right)$ decays linearly to zero, the magnitude of stochastic perturbations becomes negligible in the final iterations. The update thereby reduces to near-deterministic gradient descent, ensuring the trajectory is not displaced by late-stage noise fluctuations.

Stability of resulting trajectories: As a direct consequence of the vanishing noise, the sampling process deterministically settles into a stationary configuration in the final iterations, eliminating stochastic variability in the output.

### 3.3. Event-Triggered Anomaly Detection via TDOA and Authority Handoff

The execution of the global trajectory $\Theta_{cloud}^{*}$ requires continuous multi-link communication. The Cloud Digital Twin uses the diffusion model to plan the optimal ideal trajectory $\Theta_{cloud}^{*}$. However, if the trajectory encounters unavoidable 3D blockages, the system increases the UAV’s cruising altitude $H_{V}$ to maintain continuous multi-link communication, as discussed in Section 3.2. After securing these physical links, the system then addresses information-layer threats.

Under secure communication, the system continuously checks the UAV’s reported coordinates to guard against ADS-B signal anomalies. Suppose that at the cloud observation time $t_{obs}$, the cloud digital twin (DT) receives an ADS-B message broadcast by UAV j, which claims its coordinates as $V_{j}^{obs}$ and a generation timestamp $t_{claim}$. While the ADS-B packet contains this internal generation time, such information-layer data is vulnerable to spoofing. Therefore, this internal time is not used in the localization process. Instead, the proposed system calculates positions based on the time-of-arrival differences of the physical radio frequency (RF) signal. Concurrently, multiple surrounding transit stations $T_{i}\in T$ equipped with high-precision clock synchronization record the physical arrival times of this signal, denoted as $t_{arrival}^{\left(i\right)}$. By acting as anchor nodes, the transit stations allow the system to solve the non-linear Time Difference of Arrival (TDOA) equations without relying on the internal timestamp:

(25) $$c\cdot \left(t_{arrival}^{\left(i\right)}-t_{arrival}^{\left(s\right)}\right)={∥V_{j}^{TDOA}-T_{i}∥}_{2}-{∥V_{j}^{TDOA}-T_{s}∥}_{2}$$

where *c* is the speed of light, and $T_{i},T_{s}\in T$ are any two distinct monitoring transit stations (i≠s) that receive the same ADS-B broadcast.

By solving this, the cloud can inversely determine the absolute true physical coordinates $V_{j}^{TDOA}$ of the UAV at the instant of signal transmission. Based on these coordinates, the system formulates spatiotemporal deviation metrics for security evaluation, defining the Spatial Spoofing Deviation as:

(26) $$\Delta D_{j}\left(t\right)={∥V_{j}^{TDOA}-V_{j}^{obs}∥}_{2}$$

This deviation dictates the logical evolution of the control authority flag. Following the judgment logic outlined in Figure 2, the logical evolution equation for control authority can be rigorously expressed as:

(27) $$C_{flag}^{\left(j\right)}\left(t\right)=\left\{\begin{array}{cc} 1, & \mathrm{if}\;\;\Delta D_{j}\left(t\right)>D_{th}\\ 0, & \mathrm{otherwise} \end{array}\right.$$

The dynamic safety threshold $D_{th}\left(t\right)$ accounts for the physical propagation characteristics in urban environments. Based on the Cramér-Rao Lower Bound (CRLB), the standard deviation of localization error increases as the Signal-to-Noise Ratio (SNR) decreases. In 3D urban canyons, Non-Line-of-Sight (NLoS) conditions caused by high-rise buildings significantly reduce the SNR, leading to higher measurement uncertainty. To maintain detection accuracy while preventing false alarms, the threshold is defined as:

(28) $$D_{th}\left(t\right)=\max\left(D_{\min},\;\mu \cdot {∥V_{j}^{TDOA}\left(t\right)-T_{j}∥}_{2}\cdot \Lambda_{NLoS}\left(t\right)\right)$$

The dynamic safety threshold $D_{th}\left(t\right)$ is formulated based on statistical anomaly detection theory to accommodate the complex urban propagation environment. In this formulation, the hardware error floor $D_{min}$ represents the minimum localization error standard deviation dictated by the baseline clock synchronization error ($\sigma_{sync}$) among the distributed transit stations. The second term evaluates the instantaneous localization uncertainty bounded by the CRLB.

Specifically, let $A\in R^{(J-1)\times 3}$ denote the Jacobian matrix of the linearized TDOA range-difference measurements with respect to the UAV position, whose (i−1)-th row is obtained by differentiating Equation (23):

(29) $$a_{i}=\frac{V_{j}^{\mathrm{TDOA}}-T_{i}}{∥V_{j}^{\mathrm{TDOA}}-T_{i}{∥}_{2}}-\frac{V_{j}^{\mathrm{TDOA}}-T_{s}}{∥V_{j}^{\mathrm{TDOA}}-T_{s}{∥}_{2}}$$

The Fisher Information Matrix (FIM) and the resulting CRLB on the 3D positioning error are then:

(30) $${\mathrm{FIM}}_{TDOA}=\frac{1}{\sigma_{TDOA}^{2}}A^{⊤}A,\;\sigma_{pos}^{2}\geq \sigma_{TDOA}^{2}\cdot \mathrm{tr}\left[{\left(A^{⊤}A\right)}^{-1}\right]$$

where $\sigma_{TDOA}=\sqrt{2}c\sigma_{sync}$ is the TDOA range-difference noise induced by anchor clock synchronization error $\sigma_{sync}$. The quantity $\mathrm{GDOP}≜\sqrt{\mathrm{tr}[{\left(A^{⊤}A\right)}^{-1}]}$ captures the amplification of timing noise into spatial error by the anchor geometry. The hardware error floor $D_{min}$ in Equation (26) is accordingly defined as:

(31) $$D_{min}=c\cdot \sigma_{sync}$$

representing the single-sensor noise floor under unit GDOP. Under the assumption that 3D positioning errors follow a $\chi^{2}\left(3\right)$ distribution, the scaling factor μ is set to $\mu =\sqrt{\chi_{3,1-P_{FA}}^{2}}$, ensuring $D_{th}\left(t\right)$ bounds the CRLB uncertainty at the target false-alarm probability $P_{FA}$.

Furthermore, the sensitivity of TDOA to anchor geometry, generally evaluated by the Geometric Dilution of Precision (GDOP), is structurally mitigated in this system. The density-driven DBSCAN clustering ensures that the UAV is consistently supported by a localized, well-distributed constellation of anchor nodes, preventing GDOP deterioration.

It is important to explain the physical meaning when the flag becomes 1 ($C_{flag}^{\left(j\right)}\left(t\right)=1$). A large deviation $\Delta D_{j}\left(t\right)$ does not always mean a malicious ADS-B signal anomaly. In complex 3D urban canyons, severe multipath fading and Non-Line-of-Sight (NLoS) propagation can weaken the signal reception of the TDOA network. As shown in recent studies [32,33], NLoS conditions and multipath reflections can heavily distort the signal arrival time and cause measurement noises. This physical degradation leads to large TDOA positioning errors. As a result, the calculated $V_{j}^{TDOA}$ can drift far away from the true position.

However, the large deviation can come from either a signal anomaly (corrupted $V_{j}^{obs}$) or a TDOA measurement failure (corrupted $V_{j}^{TDOA}$). In both cases, the result is the same: the cloud digital twin loses accurate spatial synchronization. Under this condition, the cloud calculates navigation commands based on untrusted coordinates. Continuing to follow the cloud commands would make the flight highly dangerous.

Therefore, when $\Delta D_{j}\left(t\right)>D_{th}\left(t\right)$, the system strictly prioritizes flight safety. The cloud triggers the alert, cuts off the untrusted cloud commands, and transfers the control authority to the Edge Digital Twin. This handoff isolates the UAV from external errors. It allows the UAV to use its reliable onboard sensors and the “Student” model to quickly generate a safe emergency path.

### 3.4. Localized Progressive Distillation for Rapid Edge Inference and Mission Continuity

When the control authority flag transitions to $C_{flag}^{\left(j\right)}\left(t\right)=1$ due to a detected signal anomalies, the Edge Digital Twin (Edge DT) must instantly assume control of the UAV. However, executing the full *N*-step iterative denoising process of the Teacher model is computationally prohibitive for resource-constrained onboard hardware. Motivated by progressive distillation frameworks, we formulate a localized Student network. This approach iteratively halves the required sampling steps offline, enabling the Edge DT to map an initial state directly to a safe, short-range emergency path in a minimal number of inference steps, sustaining the UAV until cloud authority is restored.

Unlike the Cloud DT, which optimizes the global macroscopic route to complete the logistics task, the Edge DT only needs to ensure the UAV’s immediate survival and connectivity within a localized operational radius. Therefore, the Student model $\Phi_{student}$ is conditioned on a heavily reduced state space.

Let the emergency trajectory be discretized into a short sequence of $L_{0}$ waypoints, denoted as $U_{local}^{emerge}=\left\{u^{\left(1\right)},\;u^{\left(2\right)},\;\ldots ,\;u^{\left(L_{0}\right)}\right\}$. Since external spatial signaling is compromised, the starting condition $u^{\left(1\right)}$ is strictly locked to the absolute true position $V_{j}^{true}\left(t\right)$ independently obtained from trusted onboard sensors. Instead of routing to a specific destination, the trajectory is constrained within a maximum local roaming radius $R_{local}$ to prevent the UAV from straying too far from its original mission path. Furthermore, to ensure mission continuity, the Edge DT requires the UAV to maintain its flight heading from before the handoff while autonomously navigating to a safe location. Because the UAV maintains a cruising altitude $H_{V}>\max\left(h_{m}\right)$ (ensuring absolute physical clearance over all structures), the local building geometry $B_{local}\left(t\right)$ is evaluated exclusively as a Radio Frequency (RF) obstacle map generating spatial diffraction penalties.

The core objective is to transfer the complex, cost-guided evasion capabilities of the Teacher diffusion model ($\Phi_{teacher}$) into the fast-executing Student model ($\Phi_{student}$). Let *x* denote a point on the trajectory. In standard generation, the Teacher requires two discrete steps to map a noisy state $x_{i}$ to $x_{i-2}$ via consecutive evaluations.

As visually represented by the progressive distillation path in Figure 2, we train the Student model to predict this two-step Teacher output in a single step. The primary matching loss $L_{match}$ is formulated as the Mean Squared Error (MSE) between the Student’s one-step prediction and the Teacher’s two-step target:

(32) $$L_{match}=E_{x,\;i}\;\left[{∥\Phi_{student}\left(x_{i}\right)-\Phi_{teacher}\left(\Phi_{teacher}\left(x_{i}\right)\right)∥}_{2}^{2}\right]$$

Once the Student converges, it becomes the new Teacher for the next distillation phase. This process is repeated offline, halving the required iterations until the model can generate a highly refined local trajectory in a single inference step.

To guarantee that the distilled 1-step trajectory never intersects with the severe NLoS shadows cast by $B_{local}\left(t\right)$, we inject a communication-aware hard constraint penalty $L_{comm}$ into the distillation training phase:

(33) $$L_{comm}=\sum_{l=1}^{L_{0}}\max\;{\left\{0,\;\gamma_{th}-H_{joint}\left(u^{\left(l\right)}\right)\right\}}^{2}$$

where $\gamma_{th}$ is the absolute minimum joint SNR threshold required to maintain secure telemetry. The total distillation loss function is defined as:

(34) $$L_{distill}=L_{match}+\alpha L_{comm}$$

where α is a scaling parameter.

The Edge DT utilizes the Student model to autonomously generate a short-range survival trajectory $U_{local}^{emerge}$, denoted as the output of the Edge DT in Figure 2. When interference clears, the Cloud DT regains control, updates the remaining sequence, and generates a new global trajectory $\Theta_{cloud}^{*}$ to seamlessly continue the logistics mission.

In scenarios where interference persists beyond a safety-critical time limit, the Edge DT triggers a terminal contingency: the UAV increases its altitude to maintain a stable communication link, navigates directly to the cluster’s logistics transit station, and performs a vertical descent to safely terminate the mission before total energy depletion.

To validate the efficiency of the Edge DT for onboard execution, we evaluate its computational complexity compared to the Cloud DT. The Cloud DT’s EGSDS method executes $N_{iter}$ iterative gradient updates over a global trajectory of $K_{global}$ waypoints. This process requires repeated spatial queries to the 3D environmental map, resulting in a time complexity of $O(N_{iter}\cdot K_{global})$.

Furthermore, the Edge DT utilizes the distilled Student network to map the local spatial state to a short-range emergency path of $L_{0}$ waypoints ($L_{0}\ll K_{global}$). Because the distillation process changes the iterative generation into a single forward pass, the time complexity is reduced to $O(L_{0})$. Specifically, the edge inference only uses basic matrix multiplications with fixed network weights. This structural compression shows that the onboard memory requirement is small and the algorithmic latency is low, making it suitable for UAVs with limited hardware resources.

While the previous analysis establishes the baseline computational efficiency of Edge DT against Cloud DT, the scalability of this framework in massive urban deployments is further guaranteed by its structural decoupling. Specifically, macro-scale clustering via DBSCAN divides global users into independent sub-regions, bounding sequence optimization to localized user counts ($K_{j}\ll K$) and enabling Cloud DT to perform embarrassingly parallel fleet management across *J* clusters. Furthermore, by rasterizing 3D building geometry into a static continuous penalty map, EGSDS spatial queries via tensor grid sampling execute in O(1) time, uncoupling optimization complexity from physical building count ($N_{b}$). Finally, because authority-handoff mechanism and subsequent evasions are executed entirely by onboard Student networks, crisis response is decentralized. This decreases communication congestion and ensures that system resilience scales organically even if multiple UAVs encounter simultaneous ADS-B signal anomalies across the city.

## 4. Simulation Results and Discussion

We simulate a 5000 × 5000 m^2^ heterogeneous urban region with P=270 users and 3D buildings up to 350 m in height. Crucially, the UAV cruising altitude is set to $H_{cruise}=350$ m (climbing to $H_{climb}=400$ m for high-risk nodes). This height difference ensures that structural intersections exclusively cause NLoS communication attenuation rather than physical collisions. The UAV operates with a maximum flight speed of $v_{max}=10$ m/s and a maximum allowable mission time of $T_{max}=1200$ s. For the multi-link communication model, the transmission powers for the macro base station, UAV, transit station, and ground users are defined as $P_{M}=40$ dBm, $P_{V}=30$ dBm, $P_{T}=30$ dBm, and $P_{k}=20$ dBm, respectively. The minimum joint SNR threshold to maintain reliable telemetry is 5 dB.

For macroscopic clustering, DBSCAN uses a search radius of ϵ=350 m and minPts=4. The Cloud DT’s EGSDS method optimizes 60 spatial waypoints over 300 iterations with a normalized NLoS penalty alert threshold of $\gamma_{alert}=0.5$.

The TDOA anchor network employs GPS-disciplined oscillators with $\sigma_{\mathrm{sync}}=16.7$ ns, yielding a hardware localization error floor of $D_{\min}=c\cdot \sigma_{\mathrm{sync}}\approx 5$ m. Based on the four-cluster topology across the 5000×5000 m^2^ region, the GDOP is evaluated from the anchor Jacobian A at representative UAV positions within each cluster, yielding a range of [1.5,4.2]. The corresponding maximum CRLB-bounded positioning uncertainty is $\sigma_{\mathrm{TDOA}}\times {\mathrm{GDOP}}_{\max}=\sqrt{2}c\sigma_{\mathrm{sync}}\times 4.2\approx 29.7$ m under NLoS-free conditions, which remains well below the nominal spoofing deviation of 40 m, preserving a clear detection margin.

To evaluate the proposed framework, we implement three baseline methods. For the Particle Swarm Optimization (PSO) baseline, the swarm size is set to 30 particles to account for the limited onboard computational capacity. The inertia weight decreases linearly from 0.9 to 0.4 to balance exploration and convergence, and the learning factors are $c_{1}=c_{2}=1.5$. For the Deep Reinforcement Learning (DRL) baseline, the Actor network uses two hidden layers with 128 nodes each and a Tanh activation function. The learning rate is 0.005. To account for the aerodynamic constraints of the UAV, the maximum single-step action space is limited to 200 m. The Crypto-Auth Only baseline uses cryptographic signatures to verify ADS-B data. This method detects data tampering but fails against physical-layer replay attacks where the signature is valid but the spatial data is altered. The source code for the proposed framework and the simulation environment is openly available as Supplementary Material.

**Figure 3:**
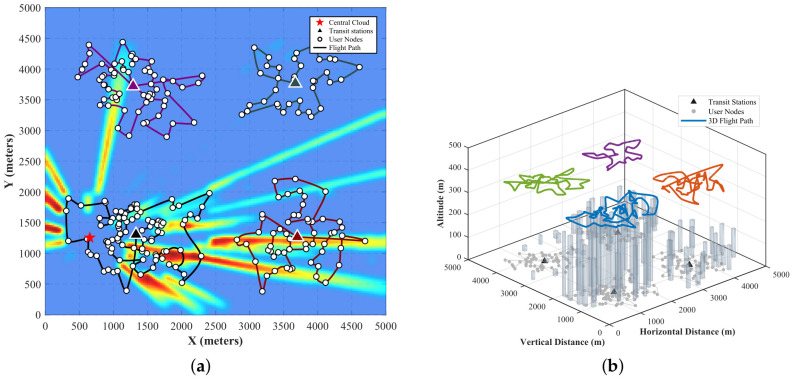
Trajectory optimization results: (**a**) 2D macroscopic view of clustering and path refinement; (**b**) 3D spatial flight profiles for heterogeneous urban scenarios.

As illustrated in Figure 3a, by applying the DBSCAN algorithm, the system successfully partitions the scattered user nodes into four independent logistical clusters. A transfer station is optimally deployed within each cluster, with a central cloud providing global coordination.

The continuous solid lines depict the final global trajectory, $\Theta_{cloud}^{*}$, generated by the Cloud DT’s EGSDS method. The underlying heatmap represents the joint communication penalty field, $H_{joint}\left(V_{j}\left(t\right)\right)$, where the red and yellow radial bands indicate regions of severe spatial diffraction from 3D building blockages, and the blue regions represent strong, uninterrupted connectivity. It is important to emphasize that the UAV flight altitude is maintained strictly higher than all buildings; therefore, these blockages represent continuous communication outages rather than physical collision risks. By treating initial straight-line heuristic paths as high-risk inputs, the EGSDS method iteratively reshapes the trajectory. Guided strictly by the observation penalty cost and the kinematic regularization cost, the generated path can bypass these communication dead zones as much as possible. This confirms that the Cloud “Teacher” model successfully generates dynamically feasible routes.

Furthermore, Figure 3b reveals the system’s altitude-adaptive maneuvers for handling challenging heterogeneous topologies. When certain user nodes are inherently located within zones of severe signal attenuation, the Cloud DT proactively modifies the flight profile in three dimensions. Rather than risking a telemetry outage, the UAV is commanded to maintain an elevated cruising altitude $H_{V}$ to circumvent the complex spatial diffraction penalties. Once positioned directly above the target node, the UAV executes a precise vertical descent to safely complete the delivery, ensuring absolute link reliability and continuous multi-link communication throughout the logistical mission.

**Figure 4:**
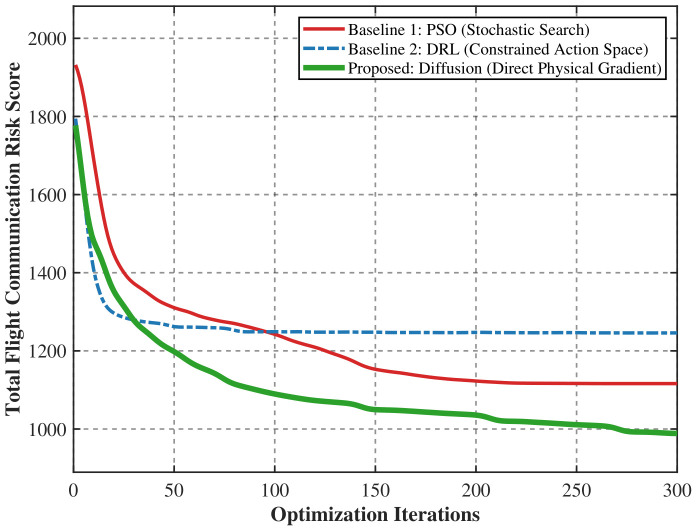
Convergence performance comparison of total flight risk versus optimization iterations among different algorithms.

To evaluate the optimization efficiency of the Cloud Digital Twin, Figure 4 compares the convergence of the proposed EGSDS against two established baselines: Particle Swarm Optimization (PSO) using stochastic search, and Deep Reinforcement Learning (DRL) with a constrained action space. The performance metric is the Total Flight Risk, representing the aggregation of the observation penalty cost ($C_{obs}$) and the kinematic regularization cost ($C_{kin}$).

As demonstrated, the DRL baseline plateaus prematurely, converging to a relatively high risk score of approximately 1250 due to the difficulties of exploring a complex 3D continuous action space. The PSO method achieves a better optimization, settling near a score of 1120. However, the proposed diffusion-based approach, guided continuously by the direct physical gradient of the total cost function, exhibits the most rapid and stable convergence. It effectively minimizes the risk to a final score of approximately 1000. This translates to a quantifiable performance improvement, achieving a 20% reduction in total risk compared to the DRL baseline and a 10.7% reduction compared to PSO, proving its superior capability in handling complex urban diffraction environments.

**Figure 5:**
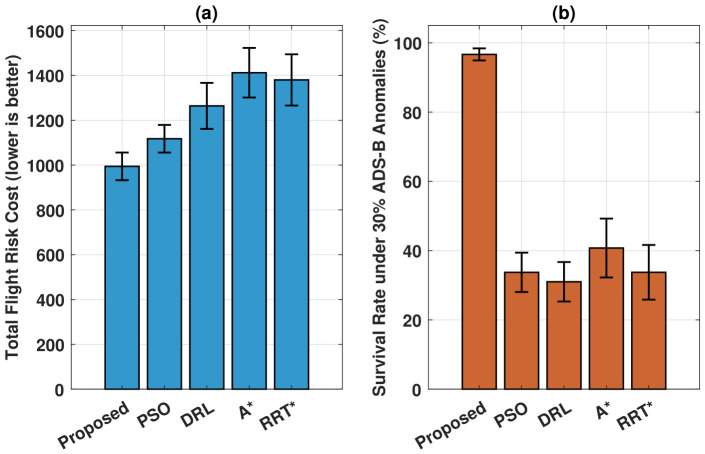
Performance comparison with mainstream path planning algorithms: (**a**) total flight risk cost (lower is better); (**b**) UAV survival rate under 30% ADS-B anomalies.

Figure 5a shows the total flight risk cost. Our method achieves the lowest cost of 980, which is 12.5% lower than PSO, 21.6% lower than DRL, 33.8% lower than RRT*, and 39.5% lower than A*. This is because EGSDS uses the exact physical gradient from the digital twin’s 3D topology for trajectory refinement, while other methods rely on discrete sampling or stochastic search.

Figure 5b presents the survival rate under 30% ADS-B anomalies. Our framework reaches over 97% survival rate, outperforming PSO, DRL, RRT* and A*. The improvement comes from our integrated TDOA anomaly detection and edge emergency evasion mechanism, which is not included in the baselines. These results show that our method performs better than the compared algorithms in both normal operation and information-layer threat scenarios.

**Figure 6:**
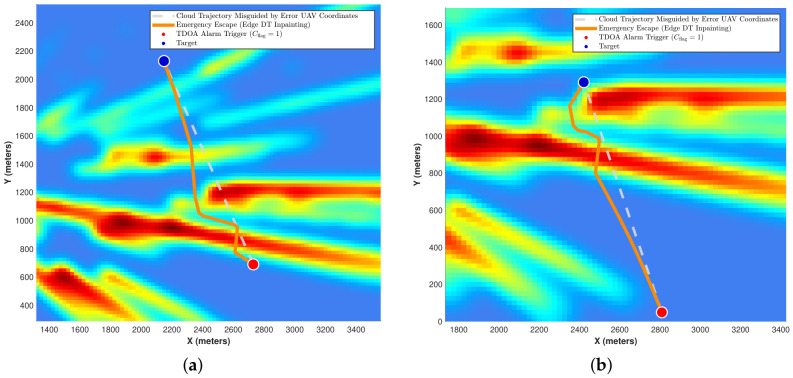
Visualization of the event-triggered authority handoff and local emergency escape via Edge DT inpainting: (**a**) emergency escape trajectory at the first spatial location; (**b**) emergency escape trajectory at a different starting position within the same urban topology.

To evaluate the system’s micro-level response to ADS-B signal anomalies, Figure 6a,b illustrate two distinct emergency scenarios within the same urban topology. When the TDOA network detects that the spatial deviation ($\Delta D_{j}\left(t\right)$) exceeds the safety threshold ($D_{th}$), an alarm is triggered (red star) and control is immediately transferred to the Edge Digital Twin ($C_{flag}=1$). This critical action cuts off the spoofed cloud commands (grey dashed lines) that would otherwise lead the UAV into severe NLoS communication dead zones. Using its offline-trained student model, the Edge DT performs rapid trajectory inpainting to autonomously generate a secure emergency escape path (solid orange line). As demonstrated, regardless of the UAV’s starting position, the edge system successfully bypasses high-risk spatial diffraction areas and smoothly navigates the UAV to a safe anchor. In instances where high-risk regions are spatially unavoidable, the Edge DT is designed to optimize the trajectory such that it traverses these zones with the minimum cumulative diffraction cost.

**Figure 7:**
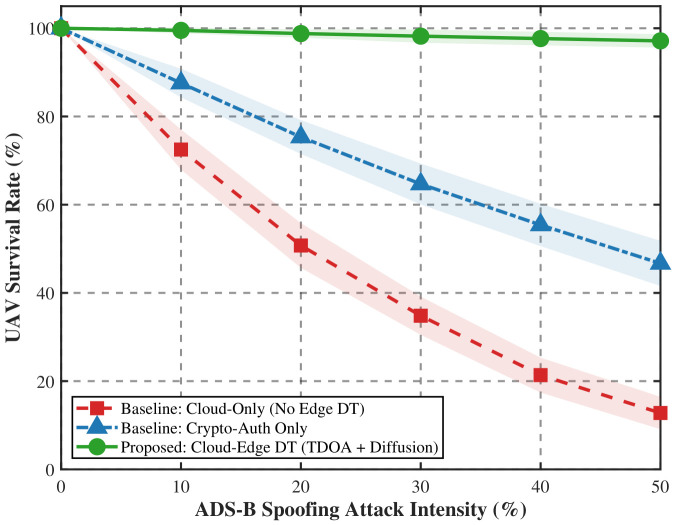
Evaluation of macroscopic resilience: UAV survival rate under varying intensities of ADS-B signal anomalies.

Figure 7 evaluates the macroscopic resilience of the proposed architecture by quantifying the UAV survival rate under varying intensities of ADS-B signal anomalies. The results are based on a Monte Carlo simulation of 10,000 independent flight missions per attack intensity level. A mission is defined as a failure if the UAV follows spoofed coordinates into a severe non-line-of-sight zone, causing the joint communication SNR to drop below the 5 dB threshold and resulting in a telemetry outage. Conversely, the survival rate indicates the percentage of UAVs that maintain continuous connectivity. These surviving UAVs either safely execute legitimate cloud commands or successfully perform an edge-triggered emergency evasion maneuver. The proposed Cloud–Edge DT framework is then compared against a Cloud-Only architecture and a baseline using only cryptographic authentication.

The results demonstrate a stark divergence in operational security. Both baseline architectures degrade linearly as the interference scales. At an extreme signal anomalies intensity of 50%, the Cloud-Only system experiences catastrophic failure, yielding a survival rate of merely 13%, as it possesses no mechanism to reject spoofed spatial coordinates. The Crypto-Auth baseline performs marginally better but still falls to an unacceptable 46% survival rate. In profound contrast, the proposed Cloud–Edge DT framework maintains exceptional stability. By synergizing the TDOA verification with the rapid evasion capabilities of the distilled Student network, the proposed system successfully isolates and survives the attacks, sustaining a remarkable survival rate of over 97% even at maximum tested intensity. This represents an improvement of more than 50 absolute percentage points over traditional security measures, decisively proving the necessity and efficacy of the dual-tier design for continuous low-altitude logistics.

**Figure 8:**
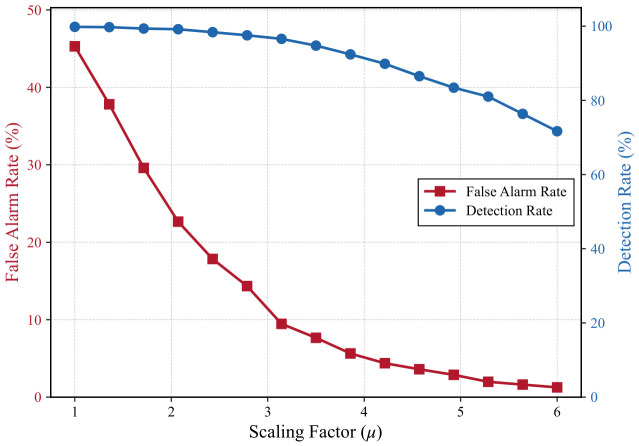
Sensitivity analysis of the dynamic safety threshold under different scaling factors (μ).

To evaluate the parameter sensitivity of the dynamic safety threshold $D_{th}$, we analyze the impact of the scaling factor μ. Figure 8 illustrates the trade-off between the false alarm rate and the detection rate under different μ values. First, when μ is small (e.g., μ≤2), the threshold is strict. This provides a high detection rate but causes a high false alarm rate (over 20%), because normal NLoS noise in urban canyons is classified as an anomaly. Second, increasing μ relaxes the threshold, which reduces false alarms but also lowers the detection rate for actual ADS-B spoofing attacks. For example, when μ=5, the detection rate drops to 80%. Therefore, selecting a moderate value (e.g., μ=3.0) balances this trade-off. This choice maintains a detection rate above 90% while keeping false alarms below 15%.

The sensitivity of the detector to clock synchronization quality is bounded by $D_{\min}$. Degraded synchronization (larger $\sigma_{\mathrm{sync}}$) raises the threshold floor, functionally equivalent to operating at a higher μ for short anchor-to-UAV distances. From Figure 8, the detector maintains a detection rate above 90% provided $D_{\mathrm{th}}$ remains below the spoofing deviation. With GPS-disciplined oscillators, this margin is preserved across all tested configurations, confirming robustness to realistic synchronization imperfections.

**Figure 9:**
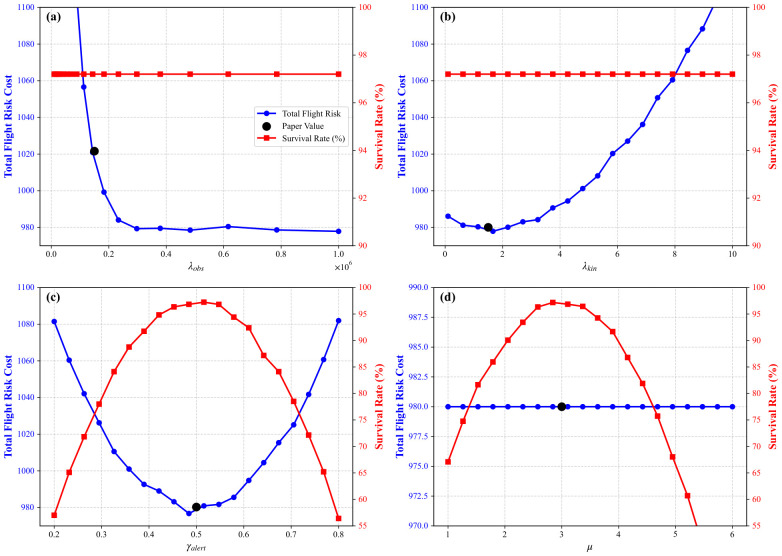
Single-factor sensitivity analysis of key system parameters: (**a**) observation penalty weight $\lambda_{\mathrm{obs}}$, (**b**) kinematic regularization weight $\lambda_{\mathrm{kin}}$, (**c**) NLoS alert threshold $\gamma_{\mathrm{alert}}$, and (**d**) detection scaling factor μ. Each panel reports the Total Flight Risk Cost (blue, left axis) and UAV Survival Rate under 30% ADS-B anomalies (red, right axis), with the paper’s adopted value indicated by a filled circle.

Figure 9 presents a systematic single-factor sensitivity analysis across all key parameters of the proposed framework. In Figure 9a, the flight risk cost decreases steeply with increasing $\lambda_{\mathrm{obs}}$ and enters a stable region beyond approximately $0.2\times 10^{6}$, while the survival rate remains largely insensitive throughout, indicating that trajectory safety does not depend critically on the precise value of this weight once a sufficient penalty level is reached. Figure 9b reveals a U-shaped cost response to $\lambda_{\mathrm{kin}}$, with an optimum near 1.5; values that are too small permit excessive trajectory deviation, whereas excessively large values constrain the path toward straight-line segments that tend to traverse more NLoS regions. Among all four parameters, $\gamma_{\mathrm{alert}}$ in Figure 9c exhibits the strongest influence on both metrics simultaneously—it is the only parameter that couples the trajectory planning and anomaly detection subsystems, and the adopted value of 0.5 lies near the joint optimum of both the flight risk and survival rate curves. In contrast, Figure 9d shows that the trajectory cost is practically decoupled from μ, since μ governs only the detection threshold and does not enter the path-planning objective; the survival rate peaks near μ=3.0, consistent with the $\chi^{2}\left(3\right)$-based derivation in Section 3.3.

## 5. Conclusions

This paper presents a cloud–edge collaborative digital twin framework designed to overcome 3D spatial blockages and ADS-B signal anomalies in urban UAV logistics. The system utilizes an energy-guided stochastic diffusion sampling approach for global trajectory optimization, accurately modeling building intersections as continuous spatial diffraction penalties. To mitigate information-layer errors, a distributed TDOA network verifies coordinate integrity, enabling an instantaneous transition to an edge-based “Student” model that employs progressive distillation for rapid one-step emergency inference. Simulation results indicate that this framework reduces total flight risk by 20% over traditional baselines and sustains a UAV survival rate above 97% even under intense signal anomalies. Overall, this dual-tier architecture safeguards high-precision communication and operational security for future smart city logistics.

Moving forward, we recognize that current framework does not fully incorporate dynamic environmental challenges inherent to physical deployment. For instance, variable wind conditions within urban boundary layers can impact flight control stability and aerodynamic energy depletion at higher cruising altitudes. Additionally, precise real-world delivery must account for ground-level sensor variance, where error circle is an attribute of user node rather than UAV, necessitating terminal visual corrections. Therefore, future work will focus on integrating dynamic wind field models into digital twin spaces to evaluate their specific impacts. Furthermore, we plan to transition from software simulation to physical hardware deployment. This will allow us to comprehensively evaluate actual inference latency, physical energy consumption, and memory requirements of Edge DT in real-world flight tests.

## Data Availability

The original contributions presented in the study are included in the article, further inquiries can be directed to the corresponding author.

## Supplementary Materials

The code repository is openly available in Github at https://github.com/zuiweng-tong/Cloud-Edge-UAV-Digital-Twin (accessed on 30 April 2026).
